# A Case Study about Joining Databases for the Assessment of Exposures to Noise and Ototoxic Substances in Occupational Settings

**DOI:** 10.3390/ijerph19084455

**Published:** 2022-04-07

**Authors:** Frédéric Clerc, Benoit Pouyatos

**Affiliations:** Pollutant Metrology and Toxicology and Biomonitoring Departments, French National Research and Safety Institute for the Prevention of Occupational Accidents and Diseases (INRS), 1 Rue du Morvan, 54500 Vandoeuvre-lès-Nancy, France; benoit.pouyatos@inrs.fr

**Keywords:** risk assessment, ototoxic substances, noise, multiple exposure assessment

## Abstract

Evaluating risks associated with multiple occupational exposures is no easy task, especially when chemical and physical nuisances are combined. In most countries, public institutions have created databases, which gather extensive information on occupational exposures or work-related diseases. Unfortunately, these tools rarely integrate medical and exposure information, and, above all, do not take into account the possible adverse effects of co-exposures. Therefore, an attempt to exploit and join different existing databases for the assessment of the health effects of multiple exposures is described herein. This case study examines three French databases describing exposures to noise and/or ototoxic chemicals (i.e., toxic to the ear) and the incidence rate of occupational deafness in different sectors. The goals were (1) to highlight occupational sectors where the workers are the most (co)exposed and (2) to determine whether this approach could confirm the experimental data showing that this co-exposure increases the risk of developing hearing loss. The results present data per occupational sector exposing workers to noise only, ototoxic chemicals only, noise and ototoxic chemicals, and neither of these two nuisances. The ten sectors in which the proportion of exposed workers is the highest are listed. This analysis shows that the rate of hearing loss in these sectors is high but does not show an increased incidence of hearing loss in co-exposed sectors.

## 1. Introduction

In most occupational settings, workers are exposed to several nuisances [1,2,3,4]. In industry, for instance, chemical exposure can be combined with high levels of noise, heat and physical burden. Evaluating the frequency and the severity of such multiple exposures for a specific occupation or, more globally, for an entire occupational sector is not an easy task. However, it can be achieved by several methods, including expert assessment, such as most job exposure matrices [5], epidemiological studies [6], surveys and surveillance data [7], or a combination of these methods [8].

Expert assessments are often conducted by public institutions, such as the French Agency for Food, Environmental and Occupational Health and Safety (ANSES), in response to requests from authorities and stakeholders. These analyses, generally conducted by scientists recruited for their knowledge, review the current data on exposures and burden of disease for specific populations, locations or occupations, and provide recommendations for the improvement of people’s safety and health. When carried out over a long period, these studies can lead to job exposure matrices, such as the Canadian carcinogen exposure surveillance project CAREX Canada [9].

This type of expertise always relies on the knowledge provided by the collected data. In France, the cross-sectional periodical survey named SUMER (French acronym for “Medical surveillance of employees’ exposure to occupational risks”) is organized every 6 or 7 years in order to collect such data at the national level. Surveillance data can also be collected by national agencies. The exposure level per nuisance data are probably the most difficult to collect. Some insights are often found in longitudinal and cross-sectional studies. Most of the time, exposure levels are only estimated and self-reported. Thus, data are often presented as ranges. Databases gathering exposure measurements in the workplace exist in different countries, such as MEGA [10], IMIS [11], COLCHIC and SCOLA [12] for chemical substances, and the Physical Agents Portal [13] or noise levels [14] for physical nuisances. In these databases, measurements are never directly linked to individuals, but are only linked to employers, machines, tasks or occupational sectors. Some organizations document occupational diseases and maintain up-to-date data. This is the case, for example, with the Bureau of Labor Statistics in the USA (https://www.bls.gov/iif/soii-data.htm, last accessed on 2 December 2021) and the “Caisse Nationale d’Assurance Maladie” (Public Health Insurance) in France (AMELI website: http://www.risquesprofessionnels.ameli.fr/last accessed on 2 December 2021). The open data provided on their websites list all occupational diseases that fall under the right to compensation and the number of cases recognized as such per occupational sector (NACE).

The ultimate goal of all these databases is to improve health and safety at work. One way to do that would be to find causal relationships between working environments and the health problems or diseases of workers. The greatest difficulty in reaching this goal is that diseases and symptoms are rarely specific to an exposure or nuisance. Some notable exceptions include bladder cancer caused by amine exposure [15] or mesothelioma due to asbestos [16]. Some toxicological relationships between exposures and diseases are demonstrated in vitro [17], in vivo in animals only [18], or in vivo in both animals and humans, but with relatively few data [19]. These findings can be convincing, but, most of the time, human data are considered more reliable than data from animal experiments. Some chemical substances found in work environments are toxic to the hearing system, i.e., ototoxic [19,20,21]. When exposure to one or several of these substances is combined with noise, then hearing impairment might be aggravated, as demonstrated by animal experiments [22] and by human observational studies [23]. Meanwhile, this has not yet been observed in existing retrospective data, and the occupational sectors where multiple exposures to noise and ototoxic substances are the most common are not well known in France and abroad.

The main ototoxic substances used in the workplace are aromatic solvents and metals. Metals can be present in inhaled air as fumes, dust or (nano)particles, or produced in cutting, grinding, polishing or welding operations. Animal data suggest that metals have distinct toxic modes of action, but all involve an oxidative stress mechanism and first target the neuronal cells and peripheral nerve fibers of the cochlea before affecting the sensory hair cells [24]. Aromatic solvents are widely used in industry for painting, varnishing, cleaning or assembling perfumes. Aromatic solvents have been the subject of numerous animal experiments over the past 20 years, and it can be considered that there is a solid scientific consensus on the toxic mechanism and the cellular targets of the members of this family. Styrene, toluene, ethylbenzene and paraxylene, to name only the most common, enter the body by inhalation or skin contact, migrating into the cochlea to poison the outer hair cells and supporting cells while sparing the inner hair cells [19].

In this case study, three French databases are joined to search for correlations and to attempt to confirm a suspected effect observed in experimental or epidemiological studies.

- SUMER provides the number and proportion of workers exposed to different nuisances for different jobs and/or occupational sectors.
- COLCHIC and SCOLA provide the intensity and/or the duration and/or the frequency of exposure to chemical substances.
- AMELI provides the yearly disease burden: the number and proportion of workers having declared an occupational disease during each calendar year.

The two aims of this case study are to portray the occupational sectors in which exposures to noise and/or ototoxic substances are the most prevalent, and to investigate if the effect of ototoxic substances and their interaction with noise can be observed in data.

## 2. Materials and Methods

### 2.1. Overview of the Proposed Methodology and the Choice of Ototoxic Substances

**Figure 1:**
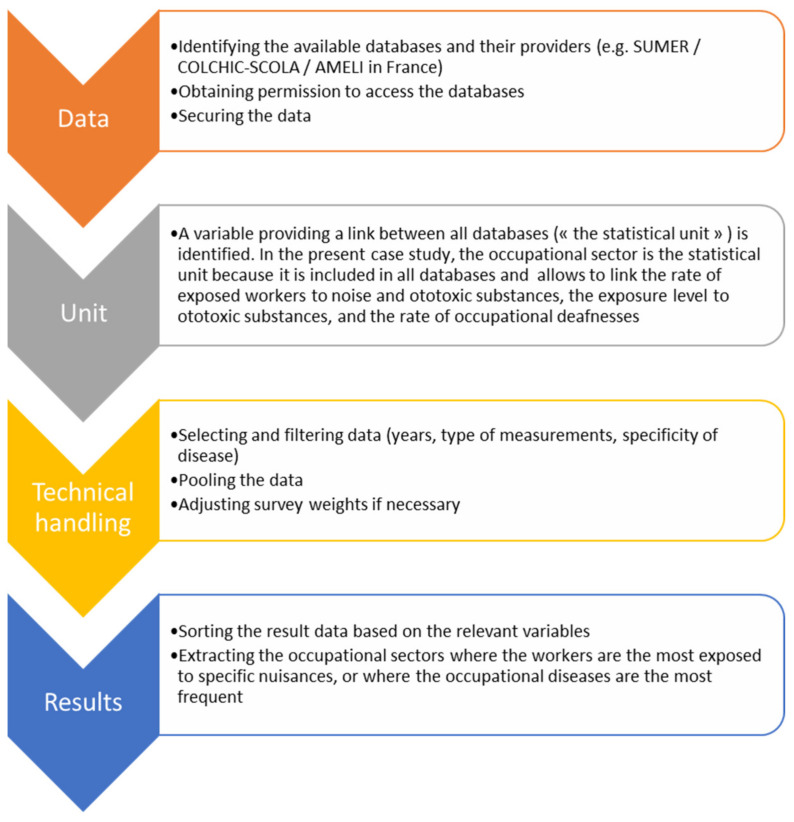
The four-step method for joining databases.

The methodology is described in Figure 1.

### 2.2. Step 1: What Are the Prerequisites and the Things to Know and to Do in Order to Obtain Access to Relevant Data?

The availability of data sources can be classified into three types. Open data are free and can be directly downloaded, or access can be easily granted by the data provider. Specific conditions of use may be imposed, such as non-commercial use or the obligation to cite sources. Public national data can often be accessed if the data request comes from an academic research institute or a university. The owner of these data might require a financial contribution or to be part of the steering committee of the study, or to be co-author of the resulting publications. In all cases, partnership conventions have to be signed between stakeholders, and this process can take months. Finally, private data, owned either by university laboratories (such as epidemiological data) or enterprises, are the most difficult to access. When access to these data is granted, the data must be hosted in a secure place in terms of software, hardware and workroom. If the data include personal nominative information, additional security measures have to be deployed, such as the traceability of data treatments or data deletion at the end of the study. Moreover, human research protections might be required, as well as related approval processes.

Four databases were used for the present case study:

- SUMER is a survey conducted every 6 or 7 years by the French Ministry of Labor. Occupational physicians collect a vast amount of data regarding workers from all occupations in France. Access to SUMER data has required a partnership. These data allow (among other things) the identification of workers exposed to noise and ototoxic substances per occupational sector. Data from the last three surveys were pooled, adjusted and used. Around 1500 variables were collected. A total of 50,000 individuals were surveyed in 2003, 48,000 in 2010 and 26,000 in 2017. These data are representative of the whole French workforce.
- COLCHIC and SCOLA are national databases hosted by the French National Occupational Health and Safety Institute (INRS), containing about 1,500,000 measurements of chemical substances at the workplace. Among these measurements collected by occupational hygienists from French public health insurance and privately held laboratories in charge of regulatory controls, the levels of exposure to eight ototoxic substances per occupational sector were extracted. Lead, styrene, toluene, n-hexane, trichloroethylene, arsenic and cobalt were selected because data were available in COLCHIC, SCOLA and SUMER, and because these substances have ototoxic properties demonstrated or suspected in humans.
- AMELI is a website edited by French public health insurance in which the number of cases of occupational hearing loss (among other pathologies) per occupational sector is provided as open data.
- Finally, the French national statistics institute (INSEE) provides the number of workers per occupational sector, also as open data.

### 2.3. Step 2: Identify or Build a Common Variable, Which Can Be Used to Link All Databases (the “Statistical Unit”)

The “statistical unit” is a variable or a set of variables shared between databases. It is also the primary key, which is a unique identifier of each data line. Therefore, the statistical unit represents the expected level of detail of the analysis. Depending on the research question, the statistical unit can be any variable, but for job exposure matrices, the job and the occupational sector are commonly used. These variables are categorical variables: they do not take ordered numbers as values, but rather as labels, often extracted from a thesaurus.

In the present case study, the occupational sector was chosen as the statistical unit. In all four databases, the data were classified using the NACE code. This code can be used at different levels of detail: there are 21 main sectors, divided into 732 occupational sectors at the most detailed level. An example of NACE codes is provided as Supplementary Materials Table S1. In this study, an intermediate level detail was chosen to work with (level 3 of the following table), which represents 272 occupational sectors.

### 2.4. Step 4: What Are the Specific Issues for the Technical Handling of the Data (Filtering, Selecting, Adjustment When Considering Survey Data, Pooling)?

There are thousands of technical issues related to data handling. In principle, the first step is to select and filter the data to remove all redundant or useless information, which might not bring meaningful knowledge to answer the research question. This step reduces the amount of data and computational time. Data from AMELI and INSEE only required typical handling, with no filtering or data transformation. By contrast, measurement data from COLCHIC and SCOLA required an important filtering process in order to focus on ototoxic substances only, as well as on individual sampling data. In addition, data analysis included the identification of the proportion of measurements above the occupational exposure limit values for each substance in each occupational sector.

When considering occupational health data, there might be a benefit of pooling different databases containing identical information. This was performed with SUMER data: the data from the three last surveys (2003, 2010 and 2017) were pooled.

SUMER data required the computation of extra variables for the identification of the number and proportion of individuals exposed to (1) noise only, (2) ototoxic substances, (3) noise AND ototoxic substances and (4) NEITHER ototoxic substances NOR noise. These extra variables were computed per occupational sector. Finally, when considering individual-based surveys such as SUMER, the data must be adjusted according to survey weights, which allow statistics to be computed upon the entire population based on the surveyed individuals. In SUMER’s case, the survey weights were provided by the data owner.

### 2.5. Step 5: The Type of Indicators Used for Communicating the Results Is Easy to Design but Is Difficult to Synthesize

The presentation of the results has to reflect the three dimensions of the analysis: the population, the exposure and the occupational disease. The indicators are expressed with regard to the chosen statistical unit: occupational sector or job. The population indicators are, at least, the number and the proportion of exposed workers. The exposure indicators reflect the nature of the nuisance or the combination of nuisances and possibly the level, the duration, or the frequency of exposure. Finally, the indicator of occupational disease is generally the incidence rate, i.e., the number of diseases per 100,000 workers of the statistical unit.

Sorting data into exposure categories is seductive. For example, “exposed/not exposed” or “high exposure/low exposure”, but the limits between these categories are often difficult to define and may result in arbitrary, unsatisfying cutoffs. Indeed, no number can properly define “exposed” versus “not exposed”. The design of statistical models is also an option for the analysis of data but may lead to complex findings that are difficult to interpret. Nevertheless, regarding the complexity and the multiplicity of data, this is probably the most reasonable way to obtain results. Finally, data visualization is definitely an issue that depends on the results.

## 3. Results: A Case Study Assessing the Multiple Exposure to Noise and Ototoxic Substances

The results of this case study are illustrated by four tables, each of them displaying the 10 occupational sectors where the largest proportion of workers have declared being exposed to noise only (Table 1); ototoxic substances only (Table 2); noise AND ototoxic substances (Table 3); and neither ototoxic substances nor noise (Table 4). For each table, statistical indicators about the populations, the exposures and the diseases are presented.

## 4. Discussion

Statistical linear models (with or without data transformations) were attempted to correlate the proportion of workers exposed to noise and ototoxic substances with the rate of occupational hearing loss. The results are not shown because no model had a satisfying predictive performance. This means the data used in this approach do not allow the observation of the toxicological findings that demonstrate the synergetic effect of noise and ototoxic substances. The major reason for this is probably related to the under-declaration of occupational hearing loss: In 2014, French health insurance estimated that about 12,500 cases of hearing loss caused by occupational noise exposures were not declared each year. This number is strikingly higher than the ~600 occupational hearing loss cases declared as caused by the occupation each year [25]. Furthermore, because exposure to ototoxic agents is not recognized as a cause of occupational deafness, workers exposed to such agents might not declare their disease if their noise exposure is low. Indeed, French labor law lists tasks and occupations that are eligible for recognition of an occupational deafness, and all of these are associated with high noise levels. In addition, the law clearly specifies that hearing loss has to be a “sensorineural hearing loss due to irreversible cochlear damage” to be financially compensated, a definition clearly associated with noise exposure. Although the presence of ototoxic substances is mentioned in the text, they are only designated as aggravating factors of the noise effect.

Furthermore, there is a lack of information about (1) the intensity of noise exposures and (2) the coding of occupational sectors. First, the only information available regarding noise exposure is a declarative statement made by the worker in the SUMER survey (“Are you exposed to noise >85 dB(A)?”). No reliable database compiling noise levels in different occupational sectors is available in France. Second, the occupational sectors used as the statistical unit may cover very different occupations and tasks and therefore may not be precise enough. The same observation was made by Cheng et al. [26].

Another limitation of the current approach is that most occupational chronic diseases appear after repeated exposure to a nuisance. The delay between the exposure and occupational disease can be counted in years, even dozens of years (asbestos mesothelioma, for example). This should be taken into account, in particular if practices or policies change the average exposure over time. For this purpose, it is important to document the role of each nuisance separately in order to estimate the probable delay between exposures and diseases. In the case of noise exposure, the delay between exposure and the occurrence of hearing loss is rather hard to define because, depending on the noise level and on the type of noise (e.g., impacts versus continuous noise), deafness can appear in hours or over the course of a working career. That is why this delay was not taken into account in this study.

More generally, the methodology is based on the assumption that there is a correlation between exposure and occupational hearing loss, as is the case, for example, in this study, which used several databases for cancer related to some occupational nuisances [27]. Therefore, a pure statistical analysis is never sufficient; toxicological and/or experimental results must be available. Such reviews of previous studies can be realized prior to the statistical analysis, as is the case in the present case study, or after the data analysis to confirm or invalidate a possibly observed statistical correlation. A consequence of this is that occupational diseases that are not linked to specific exposures or co-exposures will be difficult to detect.

The definition of “multiple exposures“ is also an issue in this type of study. The most precise approach is undoubtedly the epidemiological study: obtained data provide a direct link between the different exposures of the subjects and the adverse effect or the occupational disease. In observational data studies, such as those presented in this paper, the potential correlations between exposure and disease are presented through the aggregated statistical unit (occupational sector, job, enterprise or a combination of these). Therefore, it is impossible to precisely associate individuals with exposures; exposed individuals cannot be discriminated from unexposed ones within an occupational sector. Likewise, it is not possible to link exposures directly to diseases. The choice of the most appropriate statistical unit has a dramatic importance because a compromise between the perspectives of findings and the availability of data has to be found.

The extent of what is covered by each database must be known precisely and must be adapted (if possible) to answering the research question. In this case study, the SUMER database included individual data from the public service, whereas COLCHIC measurement data did not. In this particular case, these issues did not have an important impact because the results were presented with regard to the NACE code (statistical unit), and the public service has a specific status. This would have had an important impact if the statistical unit had included jobs that can be performed in both public and non-public service (for example, a “car mechanic”). Moreover, depending on the data sources used, the information can be more or less precise, but also possibly contradictory, so the most reliable data source for answering the research question has to be chosen. In this case study, SUMER provided information about the intensity of exposure level to chemical substances as categories (very low—low—high—very high), but COLCHIC was chosen because it provided exposure levels as airborne concentrations in mg/m^3^, which is more precise.

Finally, the technical aspects related to the data can also be improved. The visualization has to be designed from the data gathered. Based on this case study, many attempts were made to provide simple and informative figures, but none were demonstrative enough. The issue of presenting data gathered from several databases assessing multiple hazards, exposures and occupational diseases still has to be resolved. New methods of storing and processing data, such as data lakes, are promising because these methods and the associated tools allow data to be analyzed without changing them. In particular, the graph database model seems suitable since it allows connections between data from different sources to be represented [28].

Although most of the issues listed above were not taken into account in this initial analysis, current and future work will. To be effective, an analysis requires access to a wide range of data belonging to different stakeholders. Researchers, exposure scientists and occupational physicians from universities, national institutes and private companies can be involved in the gathering of data. The required data included information on exposed workers (N, repartition by age, sex), companies (size, activity sector), hazards (chemical, physical, biological, organizational, relational nuisances), level of exposure (intensity, frequency) and occupational diseases and injuries. Sharing and collaboration is therefore mandatory to perform this approach. If the data are comprehensive, such analysis of the correlations between occupational nuisances and occupational diseases is undoubtedly a meaningful way to confirm the findings of toxicological literature, and to deploy adapted occupational risk prevention actions in companies.

## 5. Conclusions

This article presents a case study in which data from different sources are joined and analyzed in order to evaluate correlations between occupational exposure to nuisances and diseases. The study focuses on exposures to noise and substances that can impair the hearing system (ototoxic substances) and their potential influence on the incidence of occupational hearing loss. The attempt to visualize the synergistic effect between noise and ototoxic agents, previously suggested by experimental studies, was not successful with this approach. This underlines the fact that the data sources used to document exposures and health effects were not comprehensive: occupational hearing loss is widely under-declared to public insurance, the delay between exposure and disease was too uncertain to be taken into account, and the average noise exposure level per sector was clearly missing. Meanwhile, the study highlighted the occupational sectors where the workers were the most exposed to the combination of noise and ototoxic substances, information that was not readily accessible. These results can be used to target the occupational sectors that require the closest attention from hygienists and occupational health services.

The method presented in this paper opens important perspectives. In particular, a joint project between database owners in France was recently launched for the analysis of multiple exposures and their correlations with occupational diseases. This project will include the development of a probabilistic approach to tackle the issue of the lag between exposure and disease.

## Figures and Tables

**Table 1:** The ten occupational sectors where the proportion of workers declaring being exposed to noise is the largest.

	SUMER					COLCHIC/SCOLA		CNAM	
Occupational Sector	Number of Workers in the Occ. Sect.	% of Workers Exposed to Noise and Ototoxic Substances	% of Workers Exposed to Ototoxic Substances Only	% of Workers Oxposed to Noise Only	% of Workers Neither Exposed to Noise nor Ototoxic Substances	Number of Measurements of Ototoxic Substances Available	% of Measurements of Ototoxic Substances above OELV	Number of Occup. Hearing loss for 100,000 Workers	Number of Occ. Hearing Loss in the Occ. Sect. Divided by nb of Overall Hearing Loss (per 100,000 Workers)
Manufacture of products of wood, cork, straw and plaiting materials	45,500	1.2%	1.7%	64.9%	33.4%	290	32%	13.9	4.4
Demolition and site preparation	85,200	1.2%	1.5%	63.8%	34.7%	223	32%	13.1	4.2
Forging, pressing, stamping and roll-forming of metal: powder metallurgy	38,900	5.6%	7.4%	59.9%	32.7%	199	38%	23.3	7.4
Construction of utility projects	61,900	1.4%	1.4%	55.1%	43.5%	37	28%	23.1	7.4
Other specialised construction activities	334,600	5.6%	7.1%	54.4%	38.5%	230	53%	10.8	3.4
Repair of fabricated metal products, machinery and equipment	74,200	3.7%	6.5%	52.8%	40.6%	133	13%	23.6	7.5
Construction of residential and non-residential buildings	130,300	1.6%	2.1%	48.0%	49.8%	85	29%	14.8	4.7
Building completion and finishing	331,200	4.3%	7.0%	47.0%	46.0%	369	35%	7.3	2.3
Treatment and coating of metals: machining	107,900	9.2%	12.2%	46.7%	41.1%	552	23%	18.7	6.0
Electrical, plumbing and other construction installation activities	374,700	5.2%	6.5%	38.3%	55.2%	71	31%	25.4	8.1

**Table 2:** The ten occupational sectors where the proportion of workers declaring being exposed to ototoxic substances is the largest.

	SUMER					COLCHIC/SCOLA		CNAM	
Occupational Sector	Number of Workers in the Occ. Sect.	% of Workers Exposed to Noise and Ototoxic Substances	% of Workers Exposed to Ototoxic Substances Only	% of Workers Oxposed to Noise Only	% of Workers Neither Exposed to Noise nor Ototoxic Substances	Number of Measurements of Ototoxic Substances Available	% of Measurements of Ototoxic Substances above OELV	Number of Occup. Hearing Loss for 100,000 Workers	Nb of Occ. Hearing Loss in the Occ. Sect. Divided by nb of Overall Hearing Loss (per 100,000 Workers)
Manufacture of medical and dental instruments and supplies	45,800	8.4%	23.3%	4.8%	71.9%	547	31.6%	2.2	0.7
Maintenance and repair of motor vehicles	118,500	11.1%	15.4%	30.0%	54.6%	497	52.3%	8.3	2.6
Manufacture of other chemical products	23,900	8.3%	15.1%	20.2%	64.7%	379	18.0%	7.0	2.2
Manufacture of basic chemicals, fertilisers and nitrogen compounds, plastics and synthetic rubber in primary forms	48,400	7.8%	12.3%	29.7%	58.0%	555	13.4%	9.6	3.1
Treatment and coating of metals: machining	107,900	9.2%	12.2%	46.7%	41.1%	552	22.8%	18.7	6.0
Manufacture of electronic components and boards	47,600	2.7%	11.2%	10.1%	78.7%	131	13.0%	1.1	0.3
Sale of motor vehicle parts and accessories	67,000	5.3%	11.0%	21.1%	67.9%	54	45.0%	3.7	1.2
Sale of motor vehicles	176,300	8.2%	10.9%	21.0%	68.1%	154	28.6%	5.2	1.7
Other specialised construction activities	334,600	5.6%	7.1%	54.4%	38.5%	230	53.2%	10.8	3.4
Building completion and finishing	331,200	4.3%	7.0%	47.0%	46.0%	369	34.7%	7.3	2.3

**Table 3:** The ten occupational sectors where the proportion of workers declaring being exposed to ototoxic substances AND noise is the largest.

Occupational Sector	Number of Workers in the Occ. Sect.	% of Workers Exposed to Noise and Ototoxic Substances	% of Workers Exposed to Ototoxic Substances Only	% of Workers Oxposed to Noise Only	% of Workers Neither Exposed to Noise nor Ototoxic Substances	Number of Measurements of Ototoxic Substances Available	% of Measurements of Ototoxic Substances above OELV	Number of Occup. Hearing Loss for 100,000 Workers	Nb of Occ. Hearing Loss in the Occ. Sect. Divided by nb of Overall Hearing Loss (per 100,000 Workers)
Maintenance and repair of motor vehicles	118,500	11.1%	15.4%	30.0%	54.6%	497	52.3%	8.3	2.6
Treatment and coating of metals: machining	107,900	9.2%	12.2%	46.7%	41.1%	552	22.8%	18.7	6.0
Manufacture of medical and dental instruments and supplies	45,800	8.4%	23.3%	4.8%	71.9%	547	31.6%	2.2	0.7
Manufacture of other chemical products	23,900	8.3%	15.1%	20.2%	64.7%	379	18.0%	7.0	2.2
Sale of motor vehicles	176,300	8.2%	10.9%	21.0%	68.1%	154	28.6%	5.2	1.7
Manufacture of basic chemicals, fertilisers and nitrogen compounds, plastics and synthetic rubber in primary forms	48,400	7.8%	12.3%	29.7%	58.0%	555	13.4%	9.6	3.1
Printing and service activities related to printing	68,900	7.7%	10.2%	20.5%	69.4%	474	23.1%	6.0	1.9
Other specialised construction activities	334,600	5.6%	7.1%	54.4%	38.5%	230	53.2%	10.8	3.4
Sale of motor vehicle parts and accessories	67,000	5.3%	11.0%	21.1%	67.9%	54	45.0%	3.7	1.2
Electrical, plumbing and other construction installation activities	374,700	5.2%	6.5%	38.3%	55.2%	71	31.3%	4.5	1.4

**Table 4:** The ten occupational sectors where the proportion of workers declaring being exposed neither to ototoxic substances nor to noise is the largest.

	SUMER					COLCHIC/SCOLA		CNAM	
Occupational Sector	Number of Workers in the Occ. Sect.	% of Workers Exposed to Noise and Ototoxic Substances	% of Workers Exposed to Ototoxic Substances Only	% of Workers Oxposed to Noise Only	% of Workers Neither Exposed to Noise nor Ototoxic Substances	Number of Measurements of Ototoxic Substances Available	% of Measurements of Ototoxic Substances above OELV	Number of Occup. Hearing Loss for 100,000 Workers	Nb of Occ. Hearing Loss in the Occ. Sect. Divided by nb of Overall Hearing Loss (per 100,000 Workers)
Residential nursing care activities	112,000	0.0%	0.0%	0.0%	100%	0	-	0.4	0.1
Monetary intermediation	315,400	0.0%	0.0%	0.2%	100%	0	-	0.0	-
Accounting, bookkeeping and auditing activities: tax consultancy	310,100	0.1%	0.2%	0.4%	99%	8	-	0.0	-
Hotels and similar accommodation	135,900	0.1%	0.1%	0.6%	99%	0	-	0.2	0.1
Management consultancy activities	152,900	0.1%	0.3%	0.5%	99%	8	-	0.1	-
Insurance, reinsurance and pension funding, except compulsory social security	656,300	0.0%	0.3%	0.9%	99%	2	-	0.1	-
Social work activities without accommodation for the elderly and disabled	245,600	0.0%	0.1%	1.4%	99%	0	-	0.4	0.1
Other human health activities	169,400	0.1%	0.5%	1.7%	98%	9	-	0.0	-
Hospital activities	523,100	0.2%	0.7%	1.5%	98%	9	-	0.3	0.1
Other social work activities without accommodation	556,300	0.0%	0.1%	2.6%	97%	0	-	0.2	0.1

## Data Availability

Chemical exposure measurements from Colchic database are not public. Any institution can be granted access on demande through www.inrs.fr (accessed on 2 December 2021). Occupational hearing loss data is publicly available from http://www.risquesprofessionnels.ameli.fr/ (accessed on 2 December 2021).

## Supplementary Materials

The following supporting information can be downloaded at: https://www.mdpi.com/article/10.3390/ijerph19084455/s1, Table S1: Example of NACE codes and their levels.
