# Supplementary material for: A Case Study about Joining Databases for the Assessment of Exposures to Noise and Ototoxic Substances in Occupational Settings
Source: Int J Environ Res Public Health. 2022 Apr 7;19(8):4455. doi: 10.3390/ijerph19084455 (PMC9032222; doi:10.3390/ijerph19084455)
Supplement: Supplementary file 1 [file ijerph-19-04455-s001.zip › ijerph-1516228-supplementary.pdf]

Table S1: Example of NACE codes and their levels

level	Code	Label
1	C	Manufacturing
2	C10	Manufacture of food products
3	C10.1	Processing and preserving of meat and production of meat products
4	C10.1.1	Processing and preserving of meat
4	C10.1.2	Processing and preserving of poultry meat
4	C10.1.3	Production of meat and poultry meat products

Example of NACE codes and its levels
